# A cyclic peptide-based PROTAC induces intracellular degradation of palmitoyltransferase and potently decreases PD-L1 expression in human cervical cancer cells

**DOI:** 10.3389/fimmu.2023.1237964

**Published:** 2023-10-02

**Authors:** Yu-Ying Shi, Di-Rong Dong, Gang Fan, Meng-Yuan Dai, Miao Liu

**Affiliations:** ^1^ Department of Gynecological Oncology, Zhongnan Hospital of Wuhan University, Wuhan, China; ^2^ Department of Urology, Huazhong University of Science and Technology Union Shenzhen Hospital, Shenzhen, China; ^3^ Department of Pathology, Brigham and Women's Hospital, Harvard Medical School, Boston, MA, United States

**Keywords:** cyclic peptide PROTAC, PD-L1, DHHC3, immunotherapy, cervical cancer

## Abstract

**Introduction:**

Our previous research has found that degradation of palmitoyltransferase in tumor cells using a linear peptide PROTAC leads to a significant decrease in PD-L1 expression in tumors. However, this degradation is not a sustained and efficient process. Therefore, we designed a cyclic peptide PROTAC to achieve this efficient anti-PD-L1 effect.

**Methods:**

We designed and synthesized an improvement in linear peptide PROTAC targeting palmitoyltransferase DHHC3, and used disulfide bonds to stabilize the continuous N- and C-termini of the peptides to maintain their structure. Cellular and molecular biology techniques were used to test the effect of this cyclic peptide on PD-L1.

**Results:**

In human cervical cancer cells, our cyclic peptide PROTAC can significantly downregulate palmitoyl transferase DHHC3 and PD-L1 expressions. This targeted degradation effect is enhanced with increasing doses and treatment duration, with a DC50 value much lower than that of linear peptides. Additionally, flow cytometry analysis of fluorescence intensity shows an increase in the amount of cyclic peptide entering the cell membrane with prolonged treatment time and higher concentrations. The Cellular Thermal Shift Assay (CETSA) method used in this study indicates effective binding between our novel cyclic peptide and DHHC3 protein, leading to a change in the thermal stability of the latter. The degradation of PD-L1 can be effectively blocked by the proteasome inhibitor MG132. Results from clone formation experiments illustrate that our cyclic peptide can enhance the proliferative inhibition effect of cisplatin on the C33A cell line. Furthermore, in the T cell-C33A co-culture system, cyclic peptides target the degradation of PD-L1, thereby blocking the interaction between PD-L1 and PD-1, and promoting the secretion of IFN-γ and TNF-α in the co-culture system supernatant.

**Conclusion:**

Our results demonstrate that a disulfide-bridged cyclic peptide PROTAC targeting palmitoyltransferase can provide a stable and improved anti-PD-L1 activity in human tumor cells.

## 1 Introduction

Although peptide drugs can fully utilize PPI (protein-protein interactions), linear peptides have conformational flexibility and are susceptible to degradation by certain enzymes, leading to inactivation (1). Certain techniques, such as stapled peptides, enhance stability and biological activity in peptides by introducing helical conformation composed of natural or non-natural amino acid residues into the peptide sequence. However, the effectiveness of stapled peptides with helical bridge links at different positions is often greatly influenced by the structure of proteins of interest (POIs) (2). Peptide cyclisation may overcome these limitations. Compared to linear peptides, the synthesis and design of cyclic peptides using disulfide bonds are considered a convenient strategy, which is believed to exhibit significant *in vivo* stability, strong resistance to proteases, and enhanced binding affinity and specificity (1, 3). These advantages make cyclic peptide compounds highly promising in drug development, with dozens of cyclic peptide drugs already entering clinical applications.

It has been proven that reducing DHHC3 (also known as ZDHHC3) to downregulate PD-L1 is feasible. However, there is a significant shortage of drugs targeting DHHC3 (4). Although reports have suggested the impact of peptide inhibitors on its function, degradation agents appear to be notably more efficient for this intracellular protein than inhibitors. Additionally, degradation agents have the potential to significantly mitigate the development of drug resistance, providing a distinct advantage in the development of DHHC3-mediated PD-L1 regulatory drugs. Previously, we developed a linear peptide PROTAC with the amino acid sequences for the binder of the von Hippel-Lindau (VHL) E3 ligase directed against PD-L1-associated palmitoyltransferase, DHHC3. This innovative design led to selective DHHC3 protein degradation in tumor cells, yielding substantial PD-L1 downregulation (5). In our pursuit of enhancing the therapeutic efficacy of this peptide, here we have created a cyclized analog. We meticulously fine-tuned the linear peptide’s sequence, converting it into a cyclic peptide PROTAC using a disulfide bridge. Our aim is to explore a novel strategy for designing tumor-targeting drug degraders by utilizing the convenient and advanced peptide drug design technique of disulfide bond-based cyclic peptides, with the goal of creating more efficient PROTAC molecules.

## 2 Materials and methods

### 2.1 Cell culture

Human cervical cancer cell line C33A was cultured in Minimum Essential Medium (Gbico) containing 10% fetal bovine serum (Invitrogen) and 1% penicillin-streptomycin antibiotics (Invitrogen). The cells were incubated at 37°C in a sterile CO2 incubator with 5% CO2. All cell experiments were performed using cells with logarithmic growth (80%- 90% fusion degree).

### 2.2 Drugs, antibodies, and inhibitors

The cyclic peptide structure is depicted in **Figure S1**, and the synthesis and purification process are as follows: Firstly, resin swelling and deprotection: Add 1g of chlorotrityl chloride (CTC) resin with a substitution degree of 0.2mmol/g to the reactor, and add 10 ml of dichloromethane (DCM). Blow dry and filter. Deprotect the resin twice. After deprotection, add 10 ml of DMF each time for resin washing, a total of 6 washes. Using solid-phase synthesis as the reaction principle, different Fmoc amino acids are added to the reactor in the same 3:1 (3eq) ratio with HOBt. Then, 10 ml of N,N-Dimethylformamide (DMF) is added, followed by the dropwise addition of 0.2 mmol (3eq) DIC. The reaction is allowed to proceed for 1 hour. Afterward, the deprotection step is performed twice. Each time, 10 ml of 20% (v/v) piperidine/DMF solution is added to the reactor. Following deprotection, 10 ml of DMF is added each time for resin washing, totaling 6 times. Continuously, amino acids are added, reacted, and synthesized in the reactor according to the known sequence. Use iodination to oxidize two cysteine residues in the peptide chain and form a cyclic structure: Dissolve the peptide in a 30% acetic acid solution and add 10 mol•L^-1^ iodine for oxidation, and let it react for 40 minutes. Then, in a centrifuge tube containing the crude peptide, add cutting solution at a ratio of 1 g resin to 10 ml cutting solution. Place it on a shaker and shake for 2 hours. Add pre-chilled ether at a 1:5 (v/v) ratio to the cutting solution, and separate the crude peptide using a low-speed centrifuge. Discard the ether into the waste container, and obtain the white solid crude peptide. Weigh the obtained solid accurately, dissolve it in 5-10% HAc solution to obtain a peptide solution with a concentration of 1 mg/ml. Use iodomethane solution as the oxidizing agent and monitor the oxidation process using mass spectrometry (MS) and high-performance liquid chromatography (HPLC). Terminate the reaction based on MS and HPLC analysis and use ascorbic acid as the terminating reagent.

Peptide purification: Use a mobile phase of 0.1% trifluoroacetic acid (TFA) water/acetonitrile for purification with a gradient of 26-43% over 40 minutes. The flow rate should be 10 ml/min, and the wavelength monitored should be 220 nm. All reagents used for peptide synthesis were procured from SynPeptide Co. Ltd (Nanjing, China). Employ HPLC for qualification control (QC) during purification, and after the sample passes the QC, perform freeze-drying to obtain a solid powder (**Figure S2**).

MG132 (IZL-3175-v) was obtained from Peptide International. The following antibodies were used in the experiments: anti-PD-L1 (ab205921) and anti-GODZ/DHHC3 (ab31837) from Abcam; rabbit secondary antibody (SA00001-2), mouse secondary antibody (SA00001-1) from Proteintech; Alexa Fluor 488-conjugated goat anti-Rabbit IgG (GB25303) from Servicebio.

### 2.3 Western blot

C33a cells were cultured and treated with the cyclic peptide at various concentrations: 0µM, 0.1µM, 0.5µM, 5µM, 10µM, 50µM, for 4 hours. A time gradient was also set at 0h, 1h, 2h, 4h, 8h, 12h, 24h, with a constant treatment concentration of 2.5µM. Total protein in each cell group was extracted using RIPA buffer (Biosharp), and the protein concentration was determined using the BCA method (Beyotime). The protein samples were mixed with 5× SDS loading buffer and denatured at 100°C for 10 minutes. Then, 20μg of denatured protein samples were subjected to electrophoresis, and the separated proteins were transferred onto a PVDF membrane. After blocking the membrane at room temperature for 2 hours, primary antibodies were incubated with the membrane overnight at 4°C. The next day, the membrane was incubated with secondary antibodies at room temperature for 1 hour. The protein bands were visualized using an ECL kit (Vazyme, E422-02) at room temperature, washed three times with 1× TBST on a shaker for 10 minutes each time, and the chemiluminescent signal was captured using a ChemiDoc XRS imaging system (Bio-Rad Laboratories Inc). The band intensity was analyzed using Image-J software, and the experiment was repeated three times to obtain an average value.

### 2.4 Cellular thermal shift assay

The treated cells were washed with PBS and detached using 0.25% trypsin from a six-well plate, followed by centrifugation. The cells were resuspended in 500μl PBS and transferred to PCR tubes. The tubes were heated at 37°C, 40°C, 43°C, 46°C, 49°C, 52°C, 55°C, 58°C, and 61°C for 3 minutes. After heating, the PCR tubes were immediately placed in liquid nitrogen for cooling, and then thawed on ice. This process was repeated three times. The tubes were then centrifuged at 4°C, 20,000 × g, for 20 minutes. Western blot analysis was performed to detect the target protein.

### 2.5 Flow cytometry

Cells were seeded in a 6-well plate with 2 mL of serum-free culture medium per well. After the cells adhered to the plate, they were treated with the cyclic peptide at different concentration gradients and time gradients. The cells were then collected by centrifugation at 1000 rpm for 5 minutes, resuspended in PBS, and protected from light. The fluorescence intensity of each group was observed and detected using a flow cytometer (Beckman), and the data were analyzed and plotted using CytExpert software.

### 2.6 Immunofluorescence

To observe the cellular distribution of the cyclic peptide and PD-L1 protein, a red fluorescence tag (rhodamine) was added to the former, and the target protein was localized using immunofluorescence staining. Cells were fixed with 4% paraformaldehyde at room temperature for 15 minutes, permeabilized with 0.5% Triton-X100 for 15 minutes, and washed with PBS. Cells were then blocked with blocking solution (1% BSA) at room temperature for 30 minutes. The cells were incubated with the primary antibody against PD-L1 (diluted 1:500 in 1% BSA) at room temperature for 2 hours. After washing with PBS in the dark, cells were incubated with the secondary antibody (diluted in 1% BSA) at room temperature for 1 hour. DAPI staining (5 μg/ml, Beyotime) was performed for 3-5 minutes to stain the cell nuclei. Anti-fluorescence quenching solution was added, and the cells were mounted. Confocal microscopy (Zeiss, LSM980) was used for observation and imaging. Care was taken to avoid exposure to light during the experiment.

### 2.7 MTT assay

C33A cells treated with the cyclic peptide were seeded in a 96-well plate at a cell density of 5 × 10³ cells per well, with 5 replicate wells for each group. After the cells adhered to the plate, the original complete culture medium was discarded, and a mixture of serum-free culture medium and MTT (Beyotime, c0009s) solution (in a ratio of 9:1) was added to each well. The plate was then incubated in a cell culture incubator for 2 hours, while being protected from light. Solutions were removed, and cells were washed with PBS three times, then 100μl of DMSO (Sigma, D2650) were added to each well and gently mixed on a shaker for 10 min at 37°. The absorbance at 490 nm for each well was measured using a microplate reader.

### 2.8 Colony formation assay

Cells treated with drugs were separately seeded in a 6-well plate and cultured continuously for 14 days. During this period, the culture medium was changed every 2-3 days. Once visible cell colonies formed, the culture medium was removed, and the cells were fixed with 4% paraformaldehyde for 30 minutes, followed by staining with 0.1% crystal violet for 30 minutes. Finally, the colonies were counted and photographed using an optical microscope.

### 2.9 ELISA

Establishment of co-culture model of cervical cancer cells and T cells: We collected peripheral blood from a 25-year-old healthy female donor and isolated human PBMCs using density gradient centrifugation. CD3 T cells were enriched using a human T cell isolation kit. The concentration of T cells was adjusted to 1*106 cells/ml and cultured with Anti-CD3 (1ug/mL), Anti-CD28 (1ug/mL), and IL-2 (10 ng/mL) for 2 days. Afterward, additional stimulation with Anti-CD3/28 and IL-2 was added, and the cells were further cultured for 2-3 days to reach the desired cell quantity for subsequent experiments. All the mentioned reagents for T cell activation were purchased from BD Pharmingen™. Cervical cancer cells treated with 10 μg/ml mitomycin were also resuspended in the culture medium and added to a 96-well round-bottom microplate in a certain concentration ratio with T cells. After incubation with different concentrations of peptide for a certain period of time, the supernatant of the cell culture was collected by centrifugation at 4°C, 350g × 5 minutes. The concentrations of the cytokines IFN-γ and TNF-α in the cell culture supernatant were determined using specific ELISA kits (nebioscience, China), according to the manufacturer’s instructions. All experiments were performed in triplicate.

### 2.10 Statistical analysis

Statistical analysis was performed using GraphPad Prism 8.0 software. All gray values and cell data were determined from at least three independent experiments and are presented as mean ± SD. Statistical significance between conditions was calculated using Student’s t-test (two-tailed) when comparing two groups; Differences between multiple means were assessed by ANOVA followed by Tukey’s *post-hoc* test. A p-value less than 0.05 was considered statistically significant.

## 3 Results

**Figure 1:**
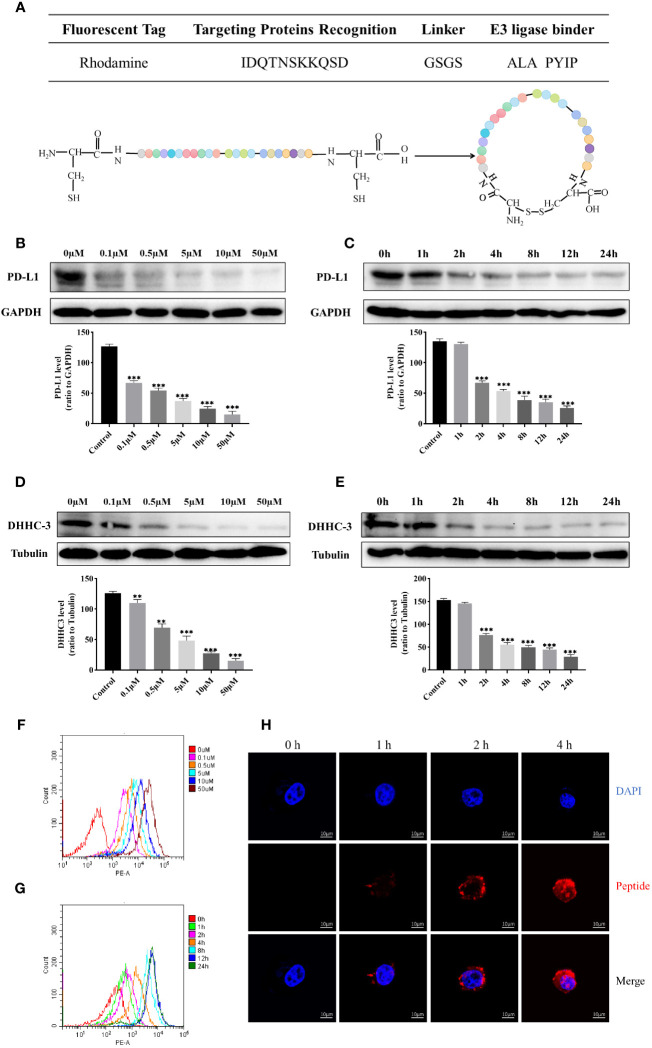
**(A)** Schematic representation of the sequence information and structural design of cyclic peptide, and the amino acid sequence “ALA PYIP” is a binder for VHL. Colored spheres represent the arrangement of amino acids as shown in the table above. **(B)** Western blot analysis of the expression of PD-L1 protein in C33A cells treated with different concentrations of cyclic peptide for 4 hours. **(C)** Western blot analysis of the expression of PD-L1 protein in C33A cells treated with 0.5 μM cyclic peptide for different incubation times. **(D)** Western blot analysis of the expression of DHHC3 protein in C33A cells treated with different concentrations of cyclic peptide for 4 hours. **(E)** Western blot analysis of the expression of DHHC3 protein in C33A cells treated with 0.5 μM cyclic peptide for different incubation times. **(F, G)** Flow cytometry analysis of fluorescent cells labeled with rhodamine after treatment with cyclic peptide at different concentrations and time gradients. Cell number was 10,000. **(H)** Confocal microscopy observation of the fluorescence intensity of cyclic peptide entering cells at different time points. Quantitative data are presented as mean ± SD, and one-way ANOVA followed by Tukey’s *post hoc* test was used for statistical analysis (n = 3): **P < 0.01, ***P < 0.001.

The characterization of the cyclic peptide was illustrated in **Figure 1A**. To investigate the changes in protein levels of PD-L1 and DHHC3 with varying concentrations and treatment durations of cyclic peptide-based PROTAC, western blot results showed a gradual decrease in the levels of PD-L1 and DHHC3 proteins as the treatment time with the cyclic peptide increased (**Figures 1B–E**). Meanwhile, the cyclic peptide had similar effects on other cancer cells such as breast cancer cell MDA-MB-231.This suggests that the targeted protein degradation has a time and concentration dependency. We also examined the effect of the cyclic peptide on PD-L1 in non-gynecological tumor cell lines (MDA-MB-231), and it exhibited a clear downregulation effect (**Figure S3**). Additionally, flow cytometry was used to measure fluorescence intensity, and the results demonstrated an increasing number of cells displaying red fluorescence with longer treatment durations and higher drug concentrations (**Figures 1F, G**). Laser confocal microscopy was employed to observe the efficiency of cyclic peptide entry into the cell membrane (**Figure 1H**), and the results indicated an increase in the amount of the cyclic peptide entering the cell membrane with longer treatment times. The DC50 values for the degradation of POIs by the drug are in the nanomolar range (**Figure S4**). In comparison to linear peptides, there is a significant enhancement in the downregulation of PD-L1. The expression of PD-L1 and DHHC3 in untreated cells remains unaltered over time (**Figures S5, S6**).

**Figure 2:**
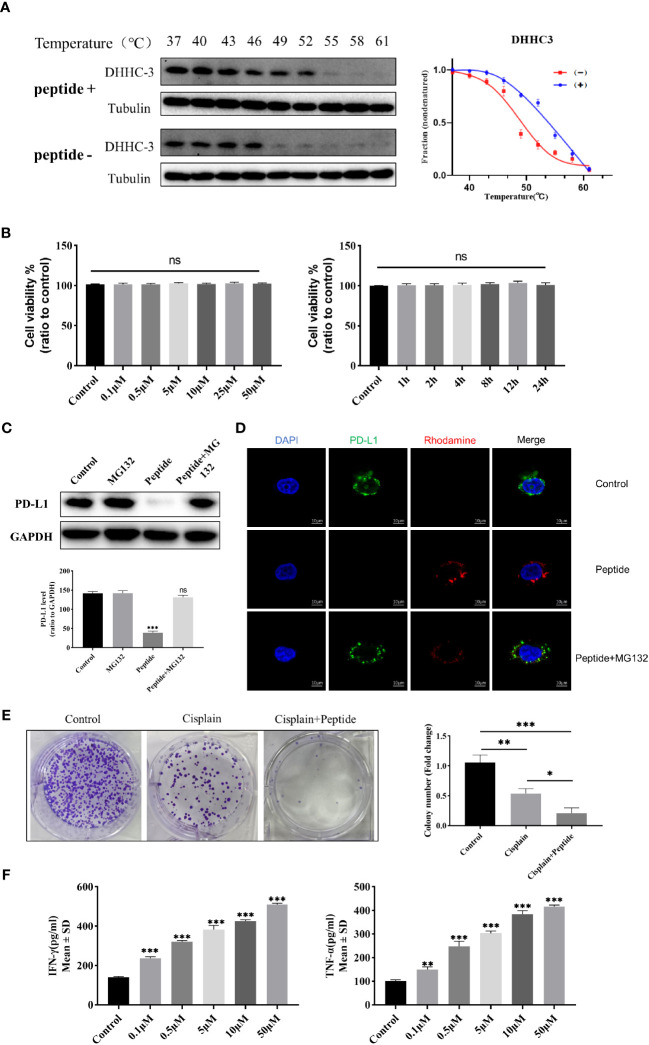
**(A)** Western blot analysis of DHHC3 protein expression levels in C33A cell lysates treated with 0.5 uM cyclic peptide at different temperatures on the left. CETSA melt curves for DHHC3 in C33A cell lysates treated with cyclic peptide on the right. Data are presented as mean ± SD of three independent experiments. **(B)** MTT assay to detect the cytotoxicity of cyclic peptide in C33A cells treated with time and concentration gradients. Data are means ± SD (n = 5). “ns” means no significant difference (p > 0.05). **(C)** Western blot analysis of PD-L1 and GAPDH protein expression levels in C33A cells pretreated with cyclic peptide alone or in combination with MG-132, for 4 hours. **(D)** Confocal microscopy observation of PD-L1 protein immunofluorescence levels with/without MG132 pretreatment. Blue, DAPI stained nuclei; Red,Cyclic peptide was labeled with rhodamine; Green, PD-L1 was labeled with antibody, detected with Alexa Fluor 488-conjugated goat anti-Rabbit IgG. **(E)** Colony formation assay to analyze the proliferation of cervical cancer cells stimulated with cisplatin and cyclic peptide. **(F)** ELISA to detect the expression levels of IFN-γ and TNF-α in the C33A-T cell co-culture model treated with different concentrations of cyclic peptide. The measurement data were represented as mean ± standard error, and one-way ANOVA followed by Tukey’s *post hoc* test was performed. *P < 0.05, **P < 0.01, ***P < 0.001.

In this study, the targeting specificity of the cyclic peptide in C33A cells was detected using the Cellular Thermal Shift Assay (CETSA) method. Western blot results showed significant degradation of DHHC3 in the untreated group at 49 degrees Celsius, while DHHC3 in the peptide-treated group exhibited substantial degradation only at 55 degrees Celsius (**Figure 2A**). The temperature-protein expression curve illustrated that the amount of DHHC3 protein in the peptide-treated group decreased more slowly with increasing temperature, and the melting curve clearly shifted to the right. Furthermore, confocal microscopy also observed the binding between the drug and DHHC3 (**Figure S7**). These results indicate effective binding between the cyclic peptide and DHHC3 protein, altering the thermal stability of DHHC3.

Furthermore, we investigated whether the cyclic peptide could enhance the degradation of PD-L1. The proteasome pathway, responsible for protein degradation, was blocked using the proteasome inhibitor MG132. Firstly, we found that PD-L1 protein levels decreased after treatment with the cyclic peptide for 4 hours at 2.5µM, and the addition of MG132 significantly reduced this degradation (**Figure 2C**). Additionally, immunofluorescence results showed a decrease in PD-L1 fluorescence intensity after the addition of the cyclic peptide, while the addition of MG132 led to a significant reversal of PD-L1 fluorescence intensity (**Figure 2D**). These results suggest that the cyclic peptide can enter the cells and induce PD-L1 protein degradation occurring through the proteasome pathway.

To assess the possible cytotoxicity of the cyclic peptide on C33A cells, we used the MTT assay to observe changes in cell viability at different time points and concentrations (**Figure 2B**). The results showed that after incubation with the cyclic peptide for up to 24 hours or at a concentration of 50µM, there was relatively minimal cytotoxicity on C33A cells, and there were no significant statistical differences observed among the different groups.

In this study, a colony formation assay was conducted to further investigate whether the cyclic peptide enhances the inhibition of C33A cell proliferation by cisplatin. From our results, compared to the blank control group, the number of C33A colonies decreased in the cisplatin-treated group, and there was a significant further reduction in the colonies formed in the cyclic peptide combined with cisplatin group (**Figure 2E**). Nevertheless, treating cells with the cyclic peptide alone did not have a significant effect on clone formation (**Figure S8**). This indicates that the cyclic peptide can enhance the inhibition of proliferation caused by cisplatin on this cell line.

We established a T cell-C33A co-culture system, where C33A cells were treated with cyclic peptide at different concentrations for 4 hours. The levels of cytokines in the supernatant were measured using ELISA. Compared to the control group, treatment with the cyclic peptide significantly increased the secretion levels of IFN-γ and TNF-α (P<0.05) (**Figure 2F**). These results indicate that the cyclic peptide targets the degradation of PD-L1, thereby blocking the interaction between PD-L1 and PD-1, and promoting the secretion of IFN-γ and TNF-α in the co-culture system into the supernatant.

## 4 Discussion

The increasing emergence of drug resistance and low efficacy of PD-1 and PD-L1 inhibitors highlights the growing significance of developing degradation agents for PD-1 and PD-L1 (6–8). While we have previously developed efficient linear peptides capable of promoting degradation of PD-1 and PD-L1, improving their *in vivo* stability remains a major challenge in our research (5). Without stable binding efficiency *in vivo*, the therapeutic potential will be greatly diminished (2). Although cyclic peptides have unique advantages in enhancing stability, not all cyclic peptides can enter cells (9). However, the peptide sequence we have optimized still exhibits good membrane permeability even after cyclization through disulfide bonds. This provides possibilities for future drug development. Furthermore, compared to our previous linear peptide results (5), the cyclic peptide utilized in this study exhibit significant inhibitory effects on PD-L1 at relatively low doses. This highly efficient antagonistic effect surpasses that of our previously reported linear peptide. Given that our cyclization-modified peptide degraders can achieve DC50 values at the nanomolar level, substantial potential for future clinical translation is evident.

Cervical cancer ranks fourth among global causes of death in women, with approximately 500,000 new cases and 300,000 deaths each year (10). Almost all cervical cancers are caused by Human papillomavirus (HPV) infection (11). Early-stage treatment for cervical cancer is relatively effective, but most patients are diagnosed in advanced stages where various treatments show limited efficacy (12). In recent years, with the flourishing development of tumor immunotherapy, the treatment options for cancer have become increasingly diverse (13). In clinical treatment for cervical cancer, PD-1 and PD-L1 antagonists are often used in combination with other treatment methods, with chemotherapy being an indispensable component of combination therapy (14, 15). Our experimental results from colony formation assay demonstrate that the combination of cyclic peptides and cisplatin has a strong inhibitory effect on cervical cancer, providing a possible new treatment solution for overcoming chemotherapy and PD-1/PD-L1 inhibitor resistance. To assess the impact of PD-L1 downregulation on T cell functionality, we established a cell co-culture system in accordance with the experimental requirements (16). The promotion of IFN-γ and TNF-α secretion in co-culture experiments of tumor cells and T cells using cyclic peptides also validates the potential of degradation agents as an effective approach to address practical issues in tumor immunotherapy, such as the low effectiveness of PD-1/PD-L1 antagonists in tumor treatment.

## Data availability statement

The original contributions presented in the study are included in the article/**Supplementary Material**. Further inquiries can be directed to the corresponding authors.

## Ethics statement

Our study was approved by the Institutional Ethics Board of Zhongnan Hospital of Wuhan University (Wuhan, Hubei, China, No. 2020029).

## Author contributions

ML conceived the project and designed the Peptide-PROTAC drugs. ML, M-YD and GF analyzed the data, and wrote the manuscript. Y-YS performed most of the experiments and statistics study. D-RD provided the assistance with the immunofluorescence experiments. All authors were involved in revising the work for intellectual content. All authors contributed to the article and approved the submitted version.
