# Supplementary material for: A cyclic peptide-based PROTAC induces intracellular degradation of palmitoyltransferase and potently decreases PD-L1 expression in human cervical cancer cells
Source: Front Immunol. 2023 Oct 2;14:1237964. doi: 10.3389/fimmu.2023.1237964 (PMC10577221; doi:10.3389/fimmu.2023.1237964)
Supplement: Supplementary file 1 [file DataSheet_1.docx]

Supplementary Materials for

**A Cyclic Peptide-based PROTAC Induces Intracellular Degradation of Palmitoyltransferase and Potently Decreases PD-L1 Expression in Human Cervical Cancer Cells.**

**
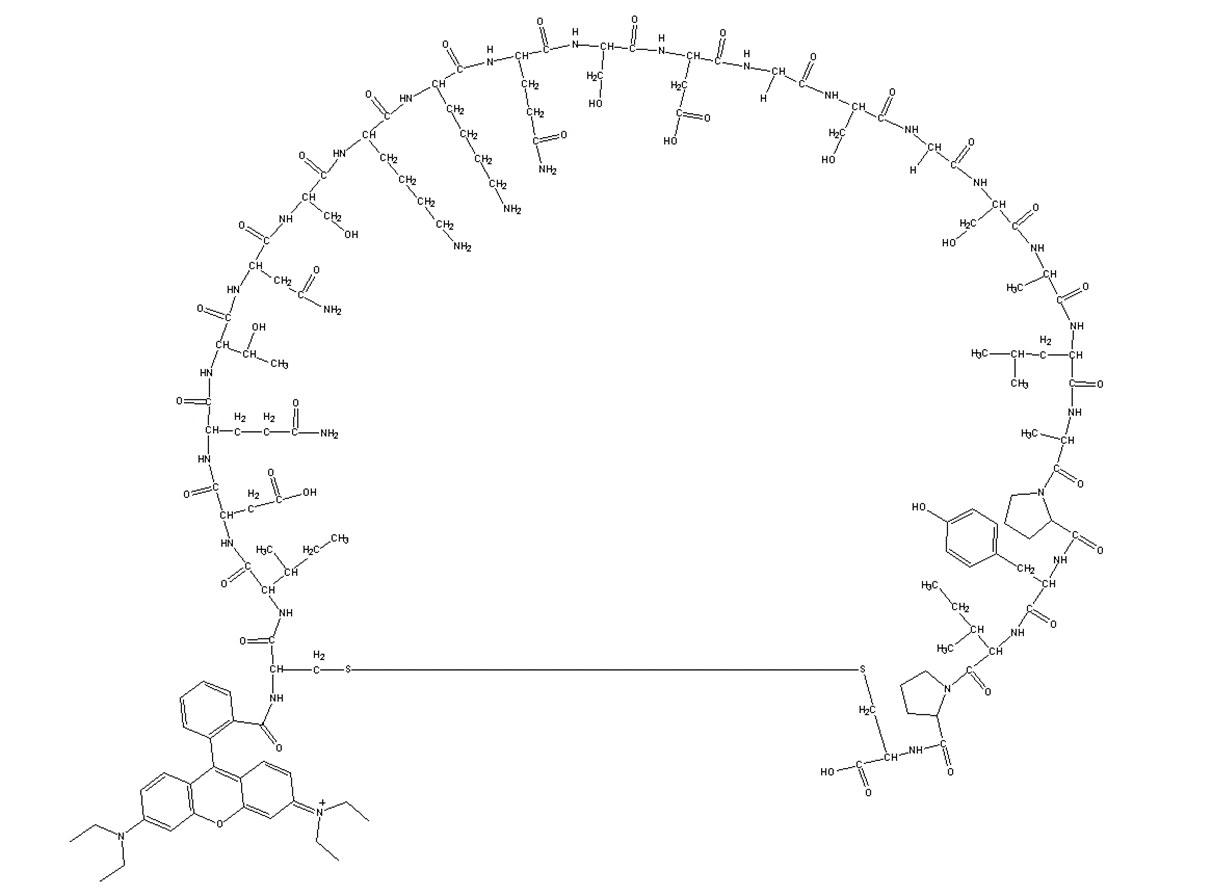
Supplementary information, Figure S1.** Cyclic peptide structure diagram

**
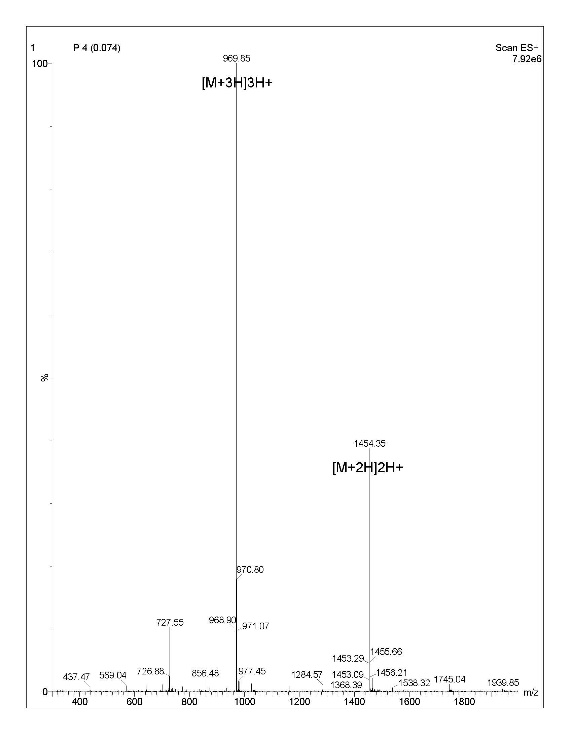

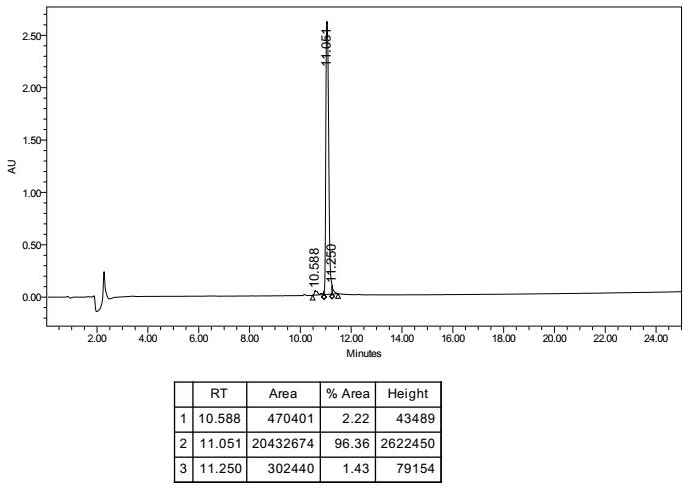
A B**

**Supplementary information, Figure S2.** Quality Control Analysis for Cyclic Peptide. (A) The peak area corresponds to the absorbance at a wavelength of 220nm in the sample during HPLC analysis RT： Retention time，the elution times of all peaks within the present analysis gradient. Area：The peak area for each individual peak % Area: The ratio of the peak's area to the total peak area; Height: The height of peak (B) Molecular weight (MW) determination: Formula for MW Calculation: Actual MW= Peak Value × Charge Number - Charge Number (Positive Ion) The theoretical MW is 2906.24, while the observed MW is 2906.55.

**
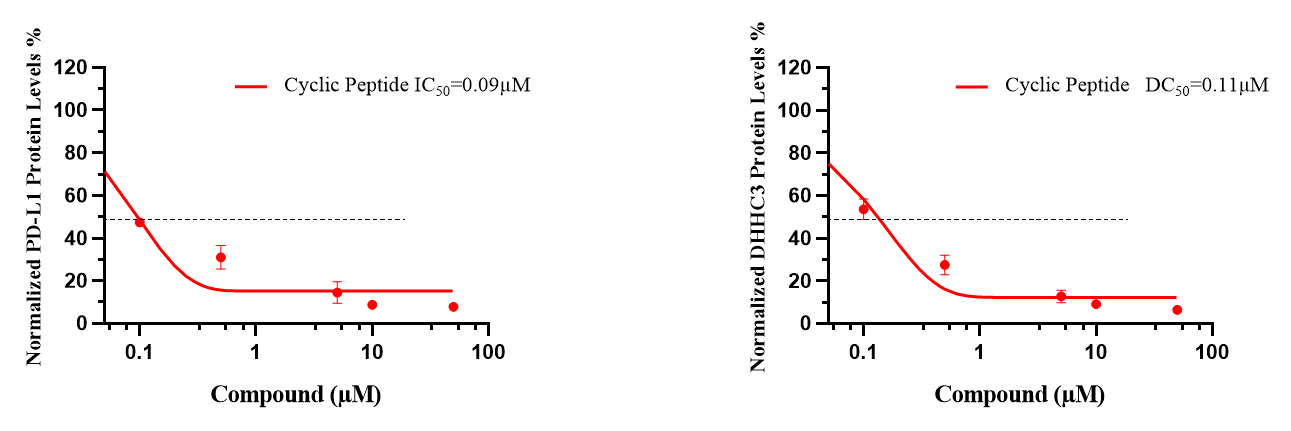
**
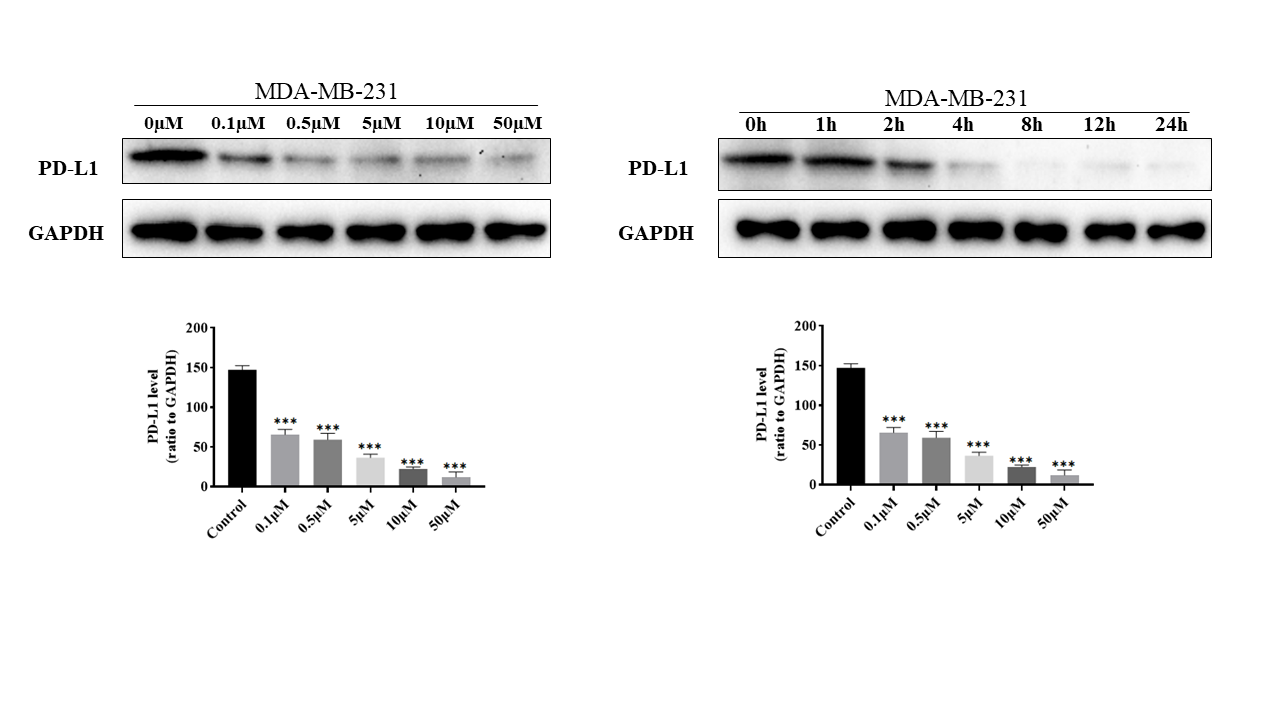
**Supplementary information, Figure S3.** Western blotting analysis shows the degradation levels of PD-L1 in the breast cancer cell MDA-MB-231 cell line after the cyclic peptide treatments for 4 h, respectively. Values are presented as mean ± SEM. One-way ANOVA followed by Tukey’s post-hoc test (n = 3): *P < 0.05, **P < 0.01, ***P < 0.001.

**Supplementary information, Figure S4.** IC50 graph of PD-L1 and DC50 graph of DHHC3 with 4 h of cyclic peptide treatment in C33A cells.

**
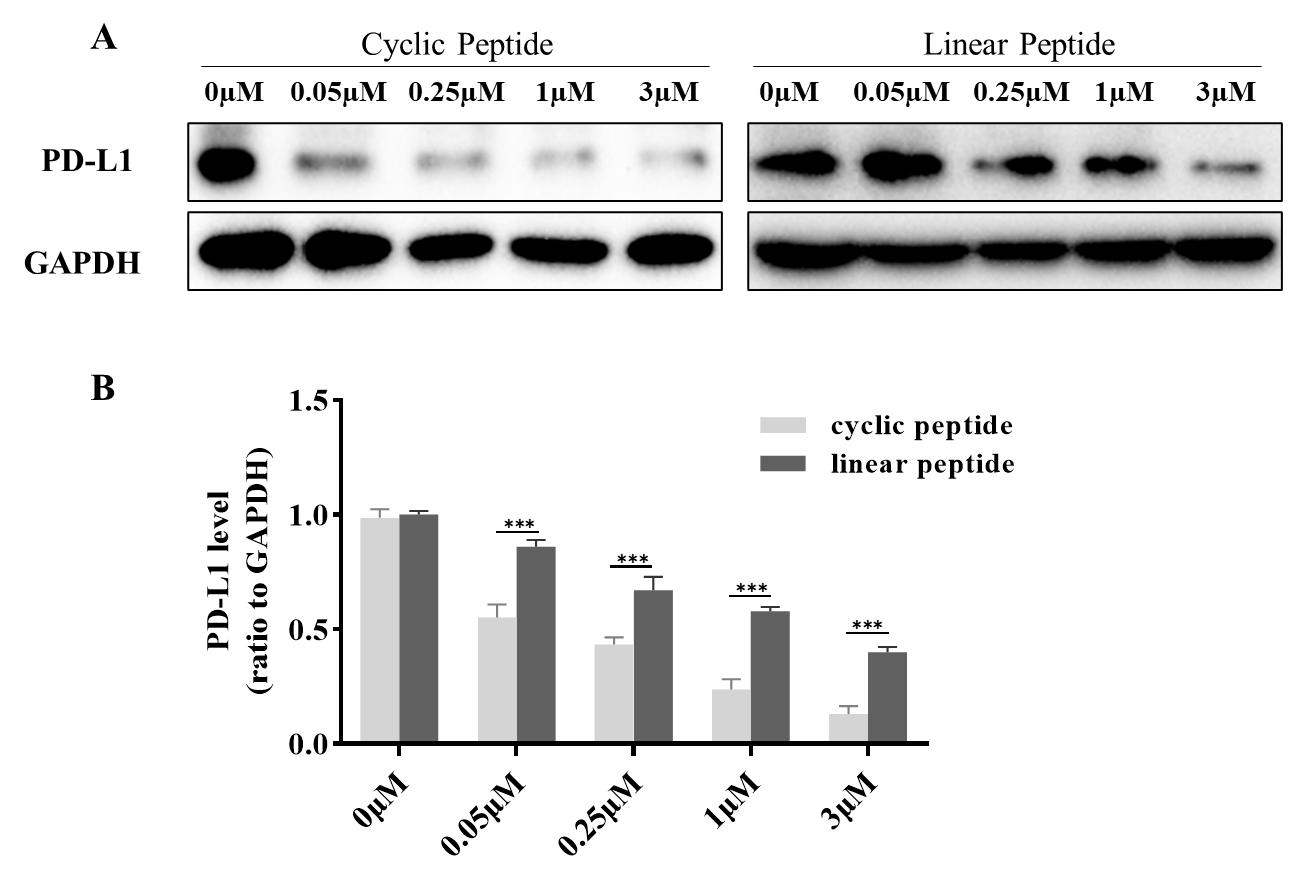
**

**
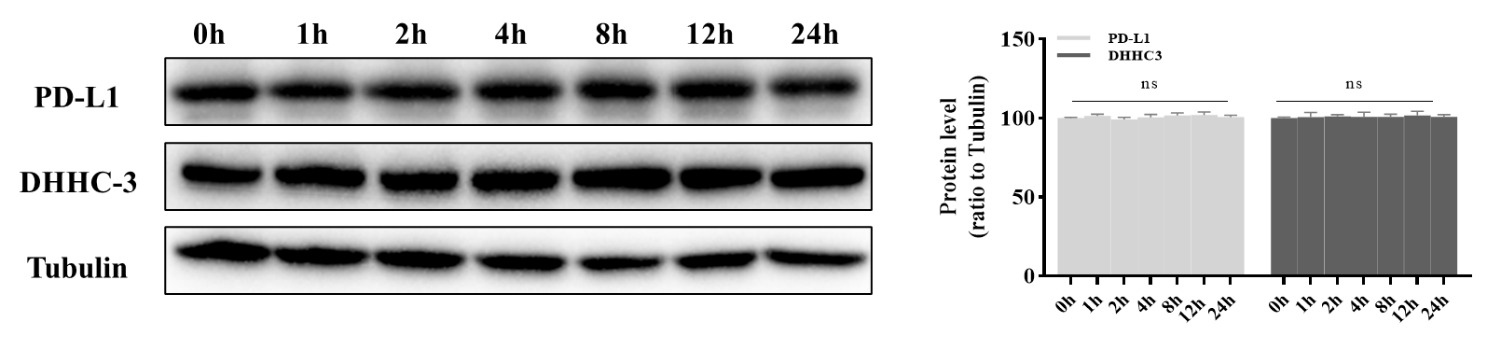
Supplementary information, Figure S5.**  A. Western blotting analysis of C33A cells after treatment with the the cyclic and linear peptide degraders at the indicated doses. B.Relative PD-L1 protein levels were quantified using the ImageJ software. Statistics, significance, ***P < 0.001.

**Supplementary information, Figure S6.** Western blotting analysis showing protein levels of PD-L1and DHHC3 in C33A cells without peptide at the indicated times. Values are presented as mean ± SEM. One-way ANOVA followed by Tukey’s post-hoc test (n = 3). ns, not significant;

**
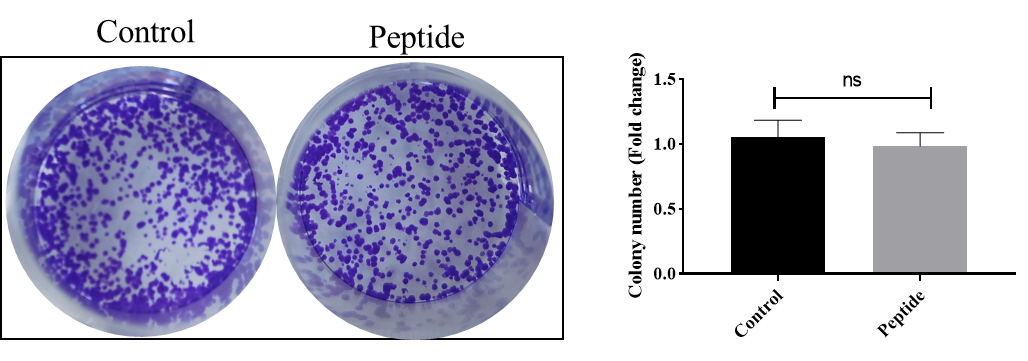

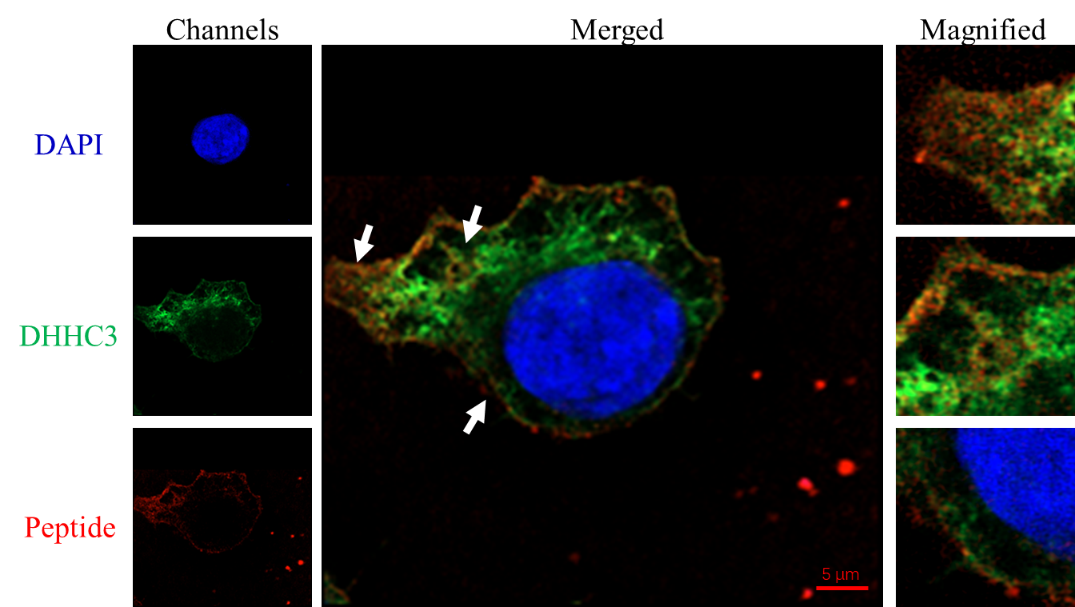
Supplementary information, Figure S7.** Confocal microscopy observation of DHHC3 protein immunofluorescence levels. Blue, DAPI stained nuclei; Red,Cyclic peptide was labeled with rhodamine; Green, DHHC3 was labeled with antibody, detected with Alexa Fluor 488-conjugated goat anti-Rabbit IgG.Scale bar, 5 μM.

**Supplementary information, Figure S8.** Colony formation assay to analyze the proliferation of cervical cancer cells stimulated with cyclic peptide. Student’s t-test when comparing two groups was performed:ns, no significance.
