# Supplementary material for: A cyclic peptide-based PROTAC induces intracellular degradation of palmitoyltransferase and potently decreases PD-L1 expression in human cervical cancer cells
Source: Front Immunol. 2023 Oct 2;14:1237964. doi: 10.3389/fimmu.2023.1237964 (PMC10577221; doi:10.3389/fimmu.2023.1237964)

Raw Data for

**A Cyclic Peptide-based PROTAC Induces Intracellular Degradation of Palmitoyltransferase and Potently Decreases PD-L1 Expression in Human Cervical Cancer Cells**

The original data is too large to upload from the upload path of the Additional files, so we generate the following link from the original data.

**Links to raw data:**

https://www.jianguoyun.com/p/DTNwGWIQpc_aCxjFzI0FIAA

**
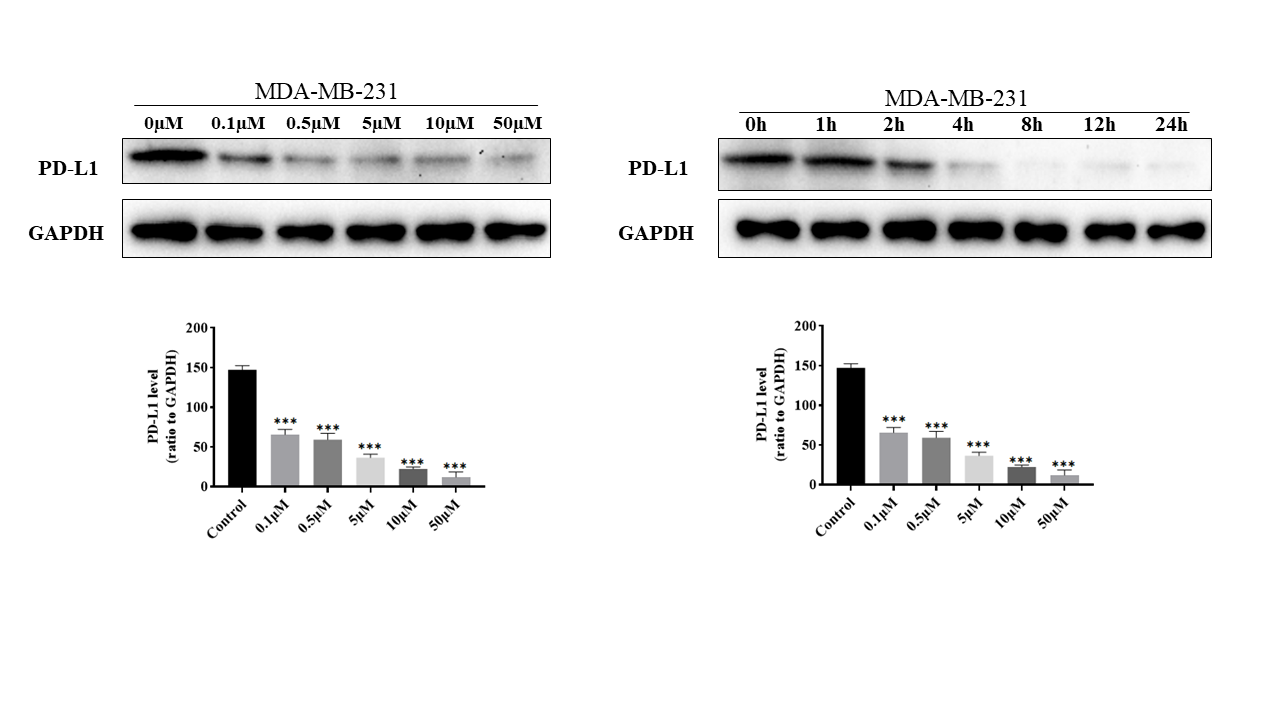
**The raw data includes four folders:

**FigureS1**

**1. Supplementary data sheet:**


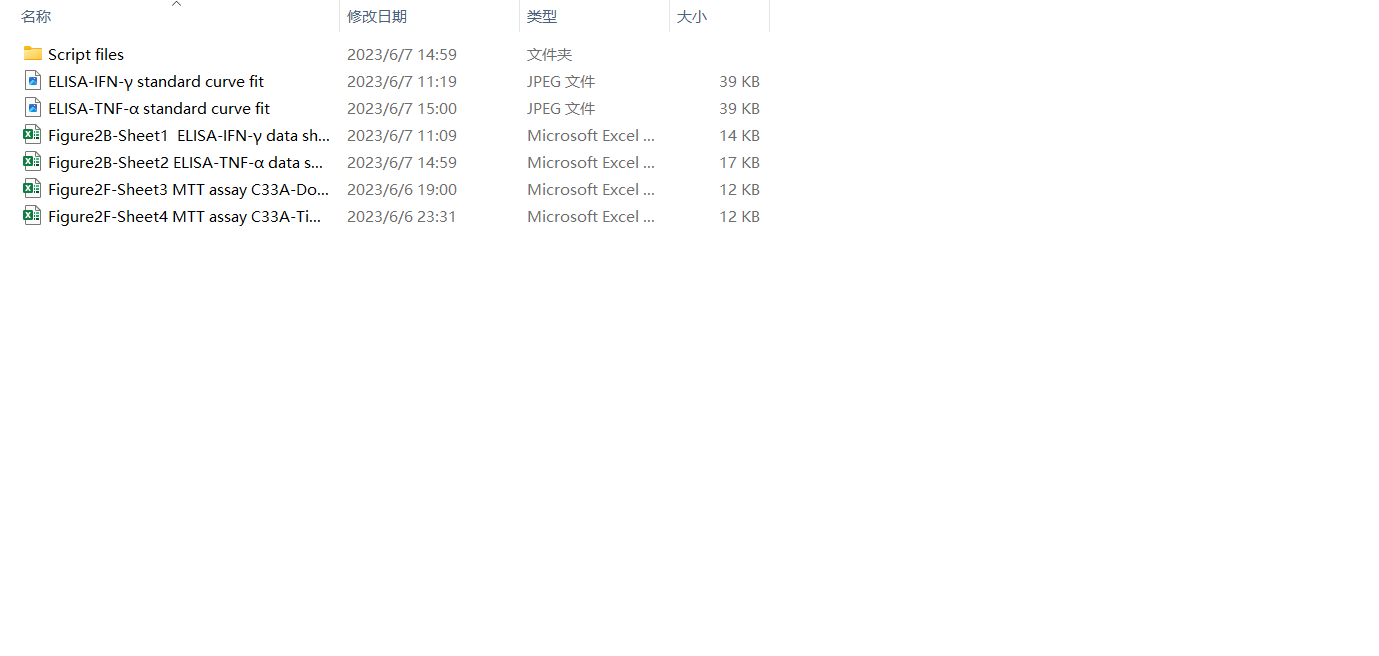
This section includes script files and data sheets for Figure 2F and Figure 2B.

The folder named as “script files” includes statistical script files used for the data analysis of ELISA and MTT assays.

**2. Supplementary flow cytometry data**

This section includes flow cytometry data (.fcs) included in Figure 1F/G.


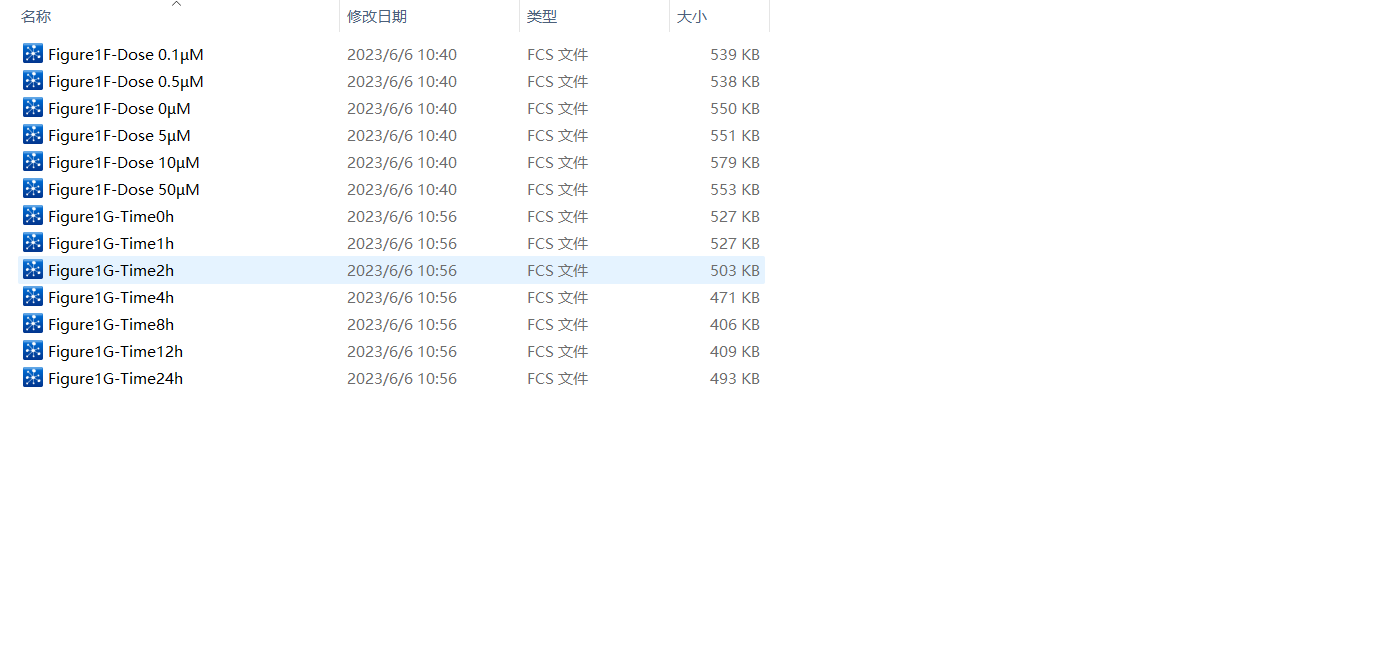


**3. Supplementary image**

This section includes five folders with images in Figure 1H and 2D.


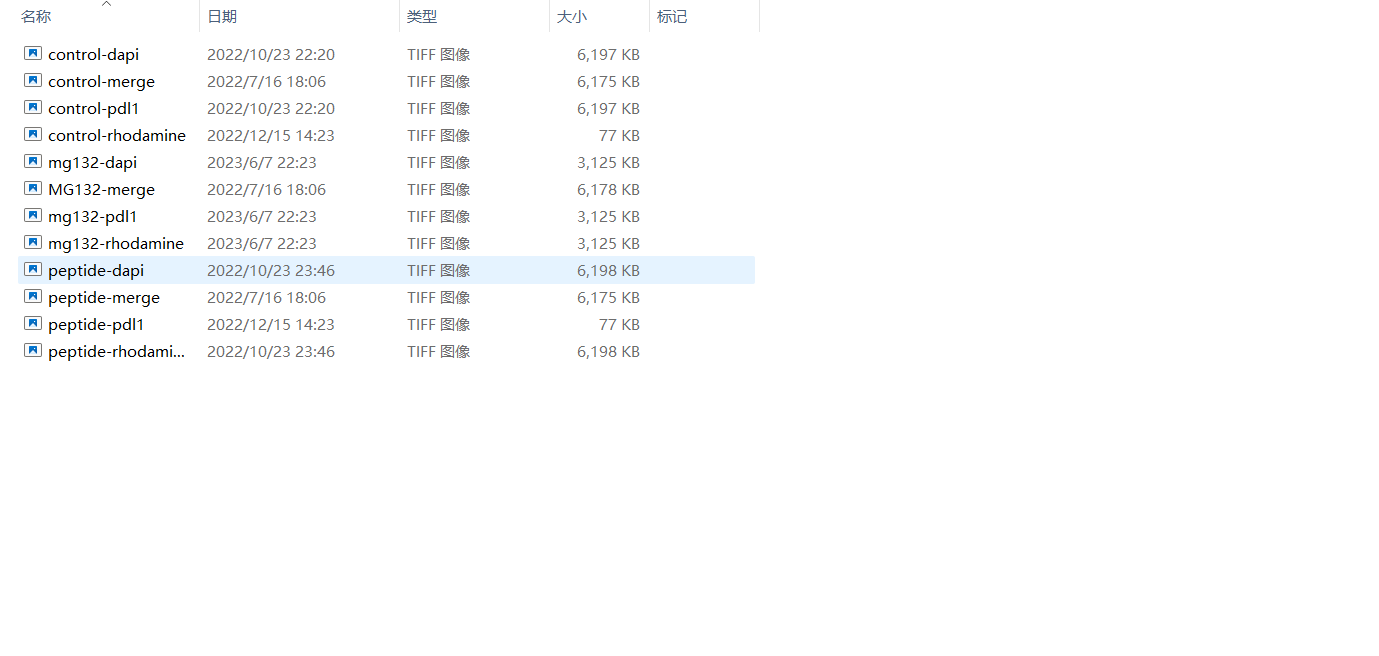

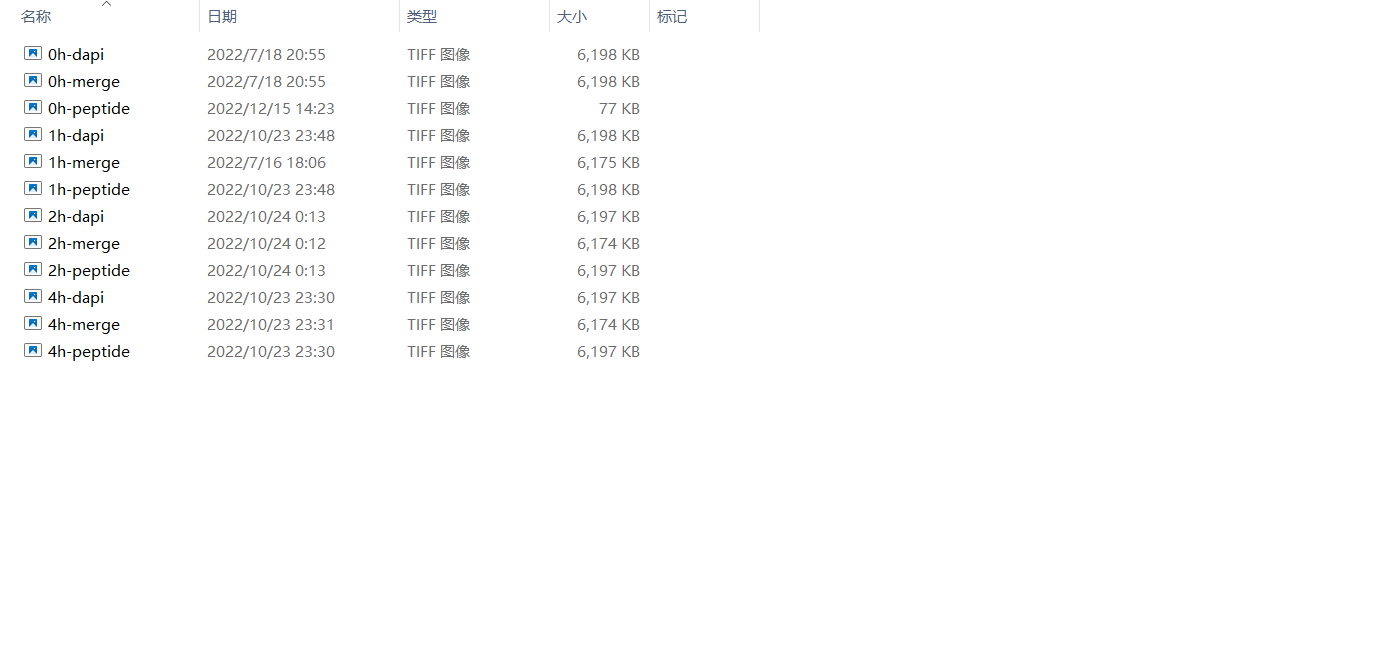


**4. Supplementary WB raw data**

This section includes Western Blot raw data in Figure 1B-E、Figure2A and 2C.


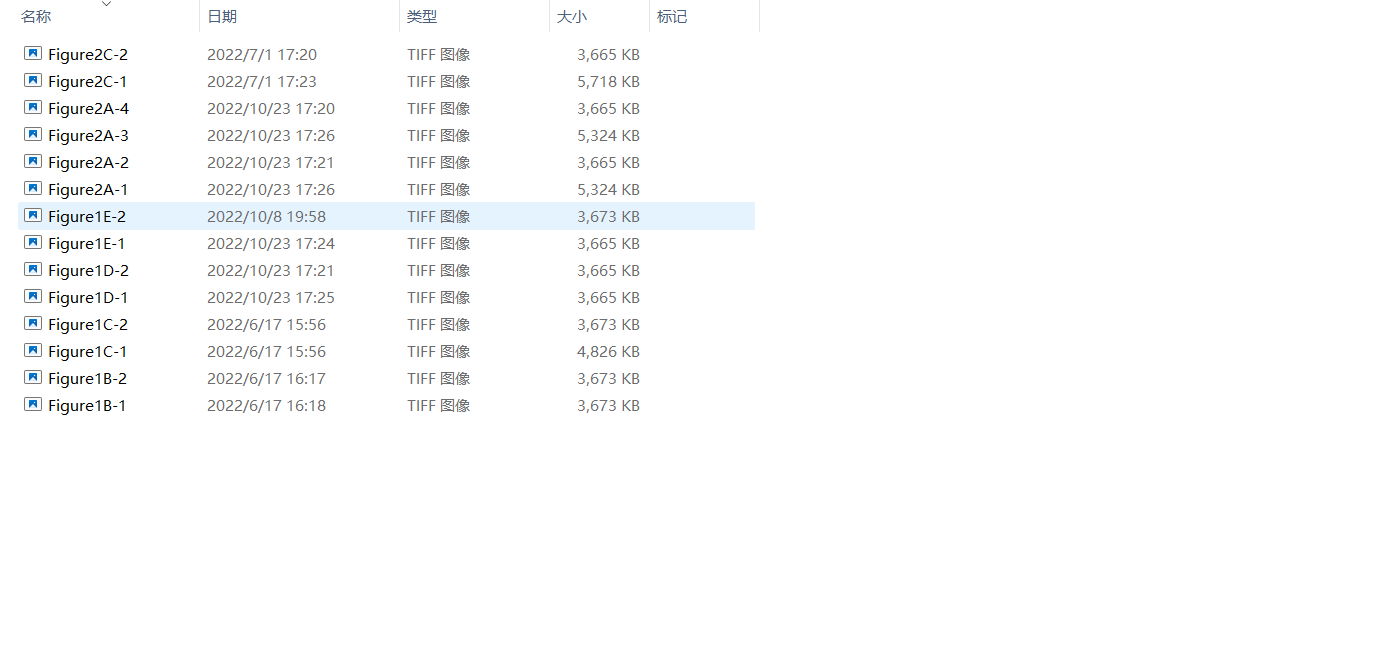

Supplement: Supplementary file 2 [file DataSheet_2.docx]
